# Characteristics of Acute Gout Flare in Patients Initiated on Intravenous Bumetanide for Acute Heart Failure Exacerbation

**DOI:** 10.7759/cureus.8605

**Published:** 2020-06-13

**Authors:** Stephanie Jeong, Irene J Tan

**Affiliations:** 1 Internal Medicine, Temple University Hospital, Philadelphia, USA; 2 Rheumatology, Einstein Medical Center, Philadelphia, USA

**Keywords:** gout, heart failure, diuretics, bumetanide, retrospective studies, allopurinol, colchicine, frequency

## Abstract

Background

Heart failure is a clinical syndrome with significant morbidity, mortality, and financial burden. These factors are magnified in patients with associated comorbidities. Therefore, addressing such conditions is critical in decreasing healthcare costs and improving patient outcomes.

Gout is a major comorbidity in patients with heart failure. Acute gout flares that occur in the context of acute heart failure exacerbations (AHFE) form an independent risk factor for increased readmissions or death. In this study, we characterized the frequency and outcomes of acute gout flares in patients treated with intravenous (IV) bumetanide for AHFE.

Methods

This single-center retrospective cohort study included 130 adult patients admitted in a tertiary-care hospital between August 2016 and June 2018. Chart review identified patients who were hospitalized for AHFE with International Classification of Diseases, Tenth Revision (ICD-10) diagnosis code I50, received IV bumetanide, and developed an acute gout flare. Data were analyzed using the chi-square test for categorical variables and the two-sample t-test for continuous variables.

Results

The annualized frequency of acute gout while receiving IV bumetanide for AHFE was 7.17%. Chronic gout patients who were on colchicine and/or allopurinol while hospitalized were less likely to develop acute gout while receiving IV bumetanide for AHFE compared with those taking neither medication (p-value =0.002). There was no significant difference in length of stay or 30-day readmissions between those who developed acute gout and those who did not.

Conclusions

Acute gout flares occur with a notable frequency in patients hospitalized for AHFE who are administered IV bumetanide. It is important to continue patients’ outpatient gout regimens in an effort to mitigate acute gout flares during this time.

## Introduction

Heart failure is a prevalent and ever-increasing public health concern that affects over five million people nationwide and is projected to cost $53 billion in healthcare expenditures by 2030 [1,2]. Symptomatic heart failure is associated with a mortality of nearly 45% at one year, portending a poorer outcome than most cancers in the United States [3]. Furthermore, in one study consisting of 1,269 patients with heart failure, 81% were found to have at least one or more comorbid conditions [4]. This was not only associated with significantly higher healthcare expenses, but also with longer hospitalization and higher mortality rates [5]. Therefore, identifying and addressing comorbidities in the context of heart failure is critical in decreasing healthcare costs and improving patient outcomes.

Gout, a clinical syndrome of joint inflammation resulting from the deposition of monosodium urate crystals, is a major comorbidity associated with heart failure [6,7]. Flares of acute gout during heart failure exacerbations form an independent risk factor for increased readmissions or death [8]. The relationship between gout and heart failure is thought to be mediated by the use of loop diuretics. Loop diuretics are known to cause increased concentrations of serum uric acid, which may precipitate new onset of gouty arthritis or cause recurrence of established gout [9]. Given the significant role that gout appears to have in the outcomes of patients with heart failure, it is important to characterize the relationship between the use of loop diuretics in treating heart failure and acute gout flares.

In this study, we aim to determine the frequency of acute gout in patients treated with intravenous (IV) bumetanide for acute heart failure exacerbations (AHFE). Secondary outcomes include length of stay (LOS) and 30-day readmissions for any cause.

## Materials and methods

**Figure 1:**
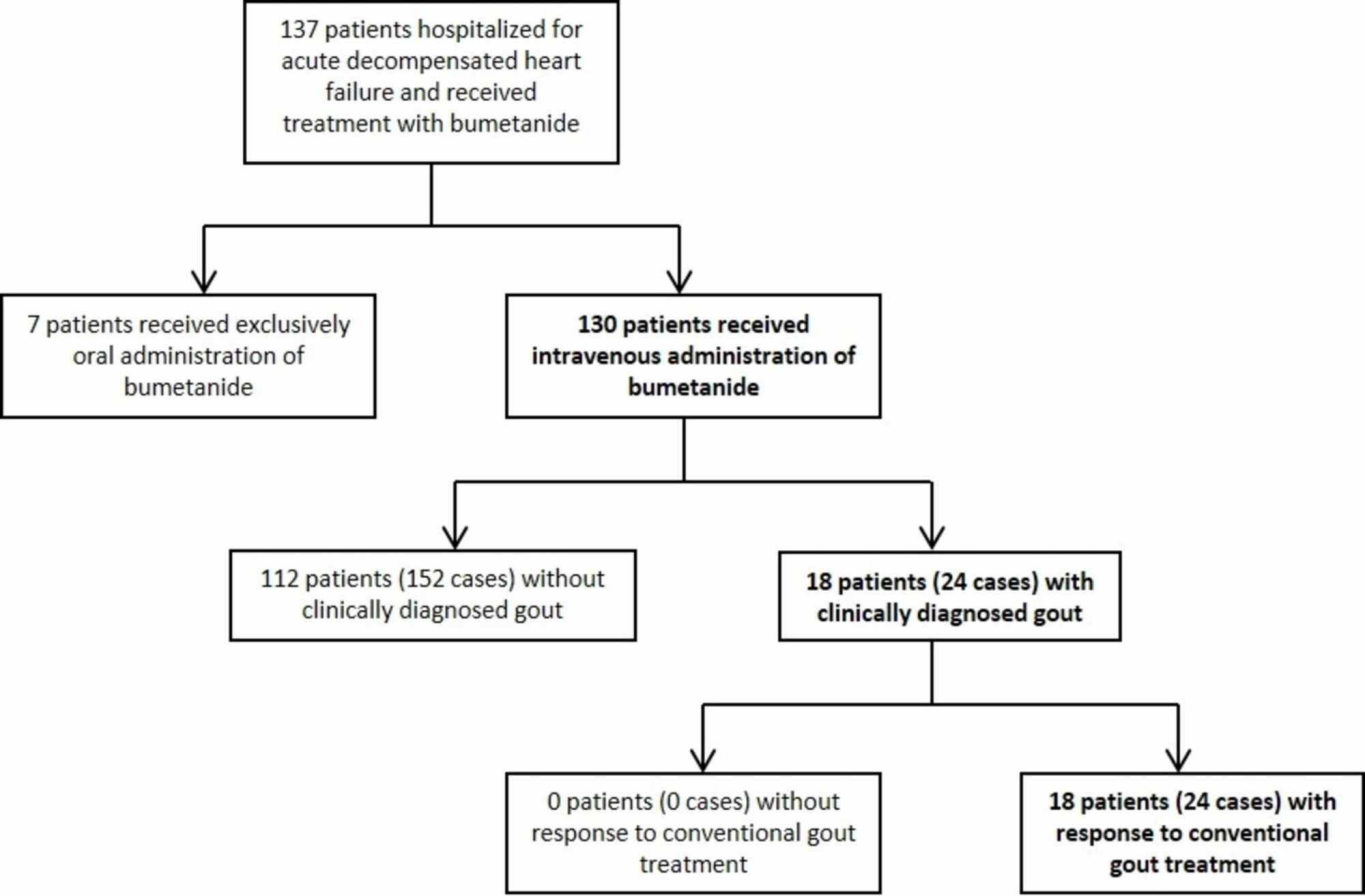
Flowchart of patient selection. Inclusion criteria were adult patients who had at least one admission for AHF exacerbation from August 5, 2016 through June 30, 2018, received intravenous bumetanide, and developed a symptomatic and clinically evident acute gout flare during the hospitalization. Patients were excluded if bumetanide was only administered orally and if there was no improvement in the presumed symptoms of acute gout despite administration of conventional treatment methods.

This single-center retrospective cohort study included adult patients admitted to an urban tertiary-care hospital between August 5, 2016 and June 30, 2018. The study was approved by Temple University Institutional Review Board protocol #25611. Chart review was performed to identify patients who were hospitalized for AHFE with International Classification of Diseases, Tenth Revision (ICD-10) diagnosis code I50, received bumetanide IV, and developed an acute gout flare. Patients were then selected through the application of specific inclusion and exclusion criteria (Figure 1).

Inclusion criteria were patients 18 years or older who had at least one admission for AHFE during this time period, received bumetanide IV at any time during hospitalization, and developed a symptomatic and clinically evident acute gout flare during the time of hospitalization. Patients were excluded from this study if bumetanide was administered exclusively by mouth and if the presumed symptoms of acute gout failed to improve despite administration of conventional treatment interventions.

Patients were identified as having an acute gout flare if the primary treating physician(s) documented a clinical picture congruous with acute gout (e.g., onset of a painful, swollen, or erythematous joint) and administered conventional treatment methods for acute gout including non-steroidal anti-inflammatory agents (NSAIDs), steroids, colchicine, urate-lowering therapies, and/or intra-articular joint injection with symptomatic improvement.

Data collected from each chart included patient demographics (date of birth, age at time of admission, gender, race, and ethnicity), admission date and diagnosis, LOS stay, bumetanide administration (including date and time of first administration, dose, and frequency), synovial fluid analysis, if obtained (including synovial white blood cell count, synovial fluid crystal identification, and fluid culture), and medical comorbidities, including known history of gout, chronic kidney disease (CKD), hypertension, diabetes mellitus, body mass index (BMI), and history of solid organ transplant (heart, lung, and kidney). Chart review was also used to identify specific medications from each pharmacologic category of gout treatment agents. NSAIDs included indomethacin, diclofenac, ibuprofen, naproxen, meloxicam, celecoxib, nabumetone, sulindac and ketorolac. Steroids included prednisone, methylprednisolone, and dexamethasone. Gout therapies included colchicine, as well as urate-lowering therapies, such as allopurinol, febuxostat, and probenecid. For patients who developed clinical signs and symptoms of gout and given conventional treatment for acute gout by their treating physician(s), the date of symptom onset, the date of administration of the acute gout treatment (NSAID, steroid, colchicine, and/or urate-lowering therapy), and the date of symptom resolution were identified. Lastly, data on readmission to the hospital for any reason within 30 days was also collected.

Data were analyzed using the chi-square test for categorical variables or the two-sample t-test for continuous variables.

## Results

A total of 137 patients were hospitalized for acute decompensated heart failure and received bumetanide between August 5, 2016 and June 30, 2018. After inclusion and exclusion criteria were applied, 130 patients were included in this study (Figure 1). These 130 patients contributed to a total of 176 cases of hospitalization for acute decompensated heart failure treated with IV bumetanide. Of these 130 patients, 18 patients developed symptoms of an acute gout flare, comprising a total of 24 cases. Of these 24 cases of clinically diagnosed gout, 18 met the 2015 American College of Rheumatology/European League Against Rheumatism (ACR/EULAR) gout classification criteria. Of the 18 patients, 12 had a known history of gout, and they contributed to 16 cases of acute gout flare. Allopurinol was the only urate-lowering therapy utilized by the treating physicians during an acute gouty flare, as none of the patients were given febuxostat or probenecid. The other 112 patients did not develop acute gout symptoms. All of the 18 patients with clinically diagnosed gout responded to conventional gout treatment. The annualized frequency of acute gout while receiving IV bumetanide for a heart failure exacerbation is 7.17%.

There was no statistical difference in age, gender, race, or BMI among patients who developed acute gout compared with those who did not develop acute gout while receiving IV bumetanide (Table 1). There was an observed non-significant trend towards developing acute gout flares in younger patients.

**Table 1:** Patient demographics including age, gender, and race. SD, standard deviation.

			Gout Symptoms		p-Value
Variable	N	Total (N=176)	Yes (N=24)	No (N=152)	
Age, mean (SD)	175	62.9 (12.8)	60.0 (10.3)	63.3 (13.1)	0.24
Gender, N (%)	175				0.65
F	80	80 (45.7%)	12 (50.0%)	68 (45.0%)	
M	95	95 (54.3%)	12 (50.0%)	83 (55.0%)	
Race	175				0.36
Black	114	114 (65.1%)	18 (75.0)	96 (63.6)	
Hispanic	32	32 (18.3%)	5 (20.8)	27 (17.9)	
White	9	20 (11.4%)	1 (4.2)	19 (12.6)	
Other	20	9 (5.1%)	0 (0.0)	9 (6.0)	

Hospitalized patients on gout therapies at the time of admission, specifically colchicine and/or allopurinol, were less likely to develop acute gout while receiving IV bumetanide for AHFE compared with those taking neither medication (p-value =0.002). Additionally, patients with a history of gout were more likely to develop an acute gout flare while receiving IV bumetanide than those without a history of gout (p-value <0.001). These findings are further described in Table 2.

**Table 2:** Development of gout symptoms by use of chronic gout therapy and history of gout.

			Gout Symptoms		p-Value
Variable	N	Total (N=176)	Yes (N=24)	No (N=152)	
Chronic gout therapy, N (%)	175				0.002
No	152	152 (86.9)	16 (66.7)	136 (90.1)	
Yes	23	23 (13.1)	8 (33.3)	15 (9.9)	
History of gout, N (%)	175				<0.001
No	124	124 (70.9)	8 (33.3)	116 (76.8)	
Yes	51	51 (29.1)	16 (66.7)	35 (23.2)	

An acute gout flare that occurred during treatment of AHF with IV bumetanide was associated with a non-significant increase in LOS by three days (p-value =0.28, Table 3).

**Table 3:** Development of gout symptoms and patient LOS. SD, standard deviation, IQR, interquartile range.

			Gout Symptoms		p-Value
Length of stay (LOS)	N	Total	Yes (N=24)	No (N=152)	
LOS, mean (SD)	175	12.1 (15.0)	15.2 (11.4)	11.6 (15.4)	0.28
LOS, median (IQR)	175	7.0 (4.0-14.0)	10.5 (7.5-25.5)	7.0 (4.0-13.0)	
LOS, median (range)	175	7.0 (0.0-120.0)	10.5 (2.0-45.0)	(0.0-120.0)	

There was no significant difference in 30-day readmissions between patients who developed acute gout and those who did not. Patients who were continued on their chronic gout therapy (colchicine and/or allopurinol) during their hospitalization for AHFE had lower 30-day readmission rates for any cause (p-value =0.013, Table 4). Those with a history of gout had higher readmission rates than those without a history of gout (p-value =0.007, Table 4).

**Table 4:** Thirty-day all-cause hospital readmission and the chronic use of colchicine or allopurinol and history of gout.

			Readmission		p-Value
Variable	N	Total (N=175)	Yes (N=95)	No (N=80)	
Development of acute gout	175				0.077
No	155	155 (88.6)	81 (85.3)	74 (92.5)	
Yes	20	20 (11.4)	14 (14.7)	6 (7.5)	
Chronic gout therapy, N (%)	175				0.013
No	152	152 (86.9)	77 (81.1)	75 (93.8)	
Yes	23	23 (13.1)	18 (18.9)	5 (6.3)	
History of gout, N (%)	175				0.007
No	124	124 (71.3)	59 (62.8)	65 (81.3)	
Yes	50	50 (28.7)	35 (37.2)	15 (18.8)	

## Discussion

Gout is a significant yet often overlooked entity that has a notable association with heart failure. Patients with AHFE frequently receive loop diuretics such as bumetanide as a part of their therapeutic management, which increases the risk of acute gout flares by elevating levels of serum uric acid [9]. Acute gout flares in these patients have been implicated as an independent risk factor for longer lengths of hospital stay, increased readmissions, or death [8]. Therefore, it is important to determine the frequency of acute gout flares in these patients as well as to discover any associated outcomes, such as LOS and readmission rates. It is also necessary to make note of any factors or interventions that may reduce the risk of developing acute gout in this patient population.

Previous studies have described this association between the use of loop diuretics and acute gout flares. In their large population-based longitudinal study, McAdams DeMarco et al. found that both loop and thiazide diuretics were independently associated with an increased risk of gout with a nine-year cumulative frequency of gout of 7% in those taking loop diuretics [9]. A systematic review and meta-analysis of prospective and retrospective cohort studies in adults by Evans et al. found that diuretic use was associated with nearly 2.5 times the risk of developing acute gout compared to no diuretic use [10].

In our retrospective cohort study, the annualized frequency of acute gout for patients receiving IV bumetanide for AHFE was found to be 7.17%. This frequency is on par with those documented in the literature [9]. However, our study only evaluated gout flares in the setting of bumetanide use, so the inclusion of other loop diuretics such as furosemide would likely yield an frequency of acute gout higher than those previously reported. Given the high burden of heart failure on both a global and national scale, this frequency should not be taken lightly. There was also found to be a significant difference in the primary outcome between patients who were continued on their chronic colchicine or allopurinol while hospitalized and those who were not. In light of this, clinicians should be aware of the importance of continuing patients’ outpatient gout regimens while managing AHFE with IV loop diuretics.

Secondary outcomes included LOS and all-cause readmission within 30 days. We found that patients who developed an acute gout flare while receiving IV bumetanide during AHFE had a longer LOS by three days compared with those who did not, though this difference was not statistically significant. However, the results surrounding readmission were more notable. First, we were able to corroborate the findings of other studies that patients with a history of gout have significantly higher readmission rates than those without a history of gout [8]. Additionally, patients who were continued on their chronic gout therapy (colchicine and/or allopurinol) during their hospitalization were found to have significantly lower 30-day readmission rates. Based on the existing literature, we suspect that allopurinol is the major player responsible for this particular finding. Uric acid is known to cause endothelial dysfunction; therefore, the reduction of uric acid with allopurinol is purported to decrease the risk of cardiovascular events [11]. A study by Thanassoulis et al. touted the use of continuous allopurinol (>30 days) in patients with gout for its ability to reduce heart failure readmissions or death and all-cause mortality [8]. Long-term high-dose (>300 mg/day) use of allopurinol was also found to have association with a better mortality than use of long-term low-dose allopurinol in patients with heart failure [11].

This study is limited by its small sample size and its single center and retrospective design. An additional limitation is that the diagnosis of acute gout, which is a central inclusion criterion for this study, was not necessarily crystal proven.

## Conclusions

In this study, we show that acute gout flares occur with a notable frequency in patients hospitalized for AHFE who are administered IV bumetanide. Patients with a known history of gout are particularly vulnerable to the development of acute gout flares in this context. The continuation of chronic colchicine and/or allopurinol yielded the opposite outcome. Further studies should investigate the use of other loop diuretics including furosemide to obtain a more comprehensive overview of incident acute gout flare in this population.
